# Fabrication of ZnO nanoparticles adorned nitrogen-doped carbon balls and their application in photodegradation of organic dyes

**DOI:** 10.1038/s41598-019-56109-3

**Published:** 2019-12-20

**Authors:** Periyasamy Thirukumaran, Raji Atchudan, Asrafali Shakila Parveen, Koteeswaran Kalaiarasan, Yong Rok Lee, Seong-Cheol Kim

**Affiliations:** 1 School of Chemical Engineering, Yeungnam University, Gyeongsan, 38541 Republic of Korea; 2 School of Material Science and Engineering, Myongji University, Yongin, Korea; 3 Center for Nanoscience and Technology, Anna University, Chennai, India

**Keywords:** Chemistry, Nanocomposites, Polymer characterization

## Abstract

In the present study, a novel ZnO nanoparticles adorned nitrogen-doped carbon balls (ZnO@CBs) were successfully synthesized from polybenzoxazine and ZnO nanoparticles through a simple carbonization method. The typical wurtzite hexagonal zinc oxide phase in ZnO@CBs and degree of graphitization were revealed by the X-ray diffraction pattern. The field emission scanning electron microscopy confirmed that the synthesized carbon materials have well dispersed ball-like structure, wherein, the ZnO nanoparticles are distributed evenly on the carbon balls (CBs). The synthesized ZnO@CBs with different wt.% (20, 40, 60 and 80) and bare ZnO nanoparticles were investigated for methylene blue (MB) dye degradation experiment. The synthesized ZnO@CBs exhibited high activity in the degradation of MB. Among the different wt.% of ZnO@CBs, 60 wt.% of ZnO@CBs showed the highest MB degradation ratio (99%) with a fast degradation rate (1.65% min^−1^) under the following optimum conditions: 20 mg of ZnO@CBs in 50 mL of MB solution at room temperature.

## Introduction

The faster population growth, industrial development, and environment transformation have emphasized the need to solve the water problem in a worldwide context and consequently, numerous systematic works have been focused to the water treatment and its sanitization by photocatalysis^1–3^. In brutality of growth and development in this field, it is required to stimulate catalyst efficiency for significant industrial applications. Until now, a large number of inorganic semiconductor materials, including metal oxides and sulfides have been used as photocatalyst for water purification. Zinc oxide (ZnO), a metal oxide semiconductor, used most extensively and intensely nowadays due to excellent optical property, electronic property, less cost and non-toxic nature. Hence, zinc oxide is considered superior than other semiconductor metal oxides.

Incapacitating of non-metals such as C, S and N, was found to reduce the band gap of metal oxides^4^. This can be achieved either by doping or coupling. In doping, these non-metals are incorporated into ZnO as dopants resulting in reduced absorption energy by producing an intermediate energy level^5^. Whereas, in coupling a hetero connection at the outer space is created with increased separation efficiency of the electron-hole pairs. So far, carbon-doped ZnO materials revealed greater optical activities^6–9^, as carbon materials are considered as the primary contender of photocatalysts materials. Lately, carbon materials, with microporosity, have been broadly accepted as the photocatalysts materials because of their high chemical and thermal stability, high surface area, outstanding electrical conductivity and low cost^10,11^. The heteroatoms such as nitrogen^12^, oxygen^13^, and phosphorus^14^ in the carbon network afford electron-donor characteristics to the carbon. Therefore, nitrogen-enriched carbons are proposed as an ideal photocatalysts material. Nowadays, nitrogen-doped carbon materials can be produced from various nitrogen containing precursors, such as polyacrylonitrile^15^, polyaniline^16^, biomass and their derivatives^17^. Polybenzoxazine (PBz), a polymer produced from benzoxazine monomer and also a nitrogen-containing polymer is considered to be a promising source of carbon due to its outstanding thermal properties, high char yield and excellent molecular design flexibility^18^. PBz resins can be effortlessly synthesized from phenols, amines, and formaldehyde by a low-cost method. Nevertheless, there are only few works reported on the synthesis of nitrogen-doped carbon materials derived from PBz^19–21^. The excellent thermal and chemical stability of PBz makes them retain the high content of nitrogen and oxygen species in their carbon structure even after carbonization and activation of the precursors. Hence, choosing PBz as the ideal source of carbon precursor is an admirable way of producing hetero-doped carbon to be used particularly in photocatalytic applications. Therefore, PBzs could be considered as an ideal precursor of photocatalyst materials for dye degradation.

The present work reports on the facile synthesis of ZnO nanoparticles adorned nitrogen-doped carbon balls (ZnO@CBs) derived from polybenzoxazine. Solution method was adopted to synthesize benzoxazine monomer from eugenol, ethylenediamine and formaldehyde. ZnO@CBs was synthesized by means of calcination (carbonization) approach with the use of ZnO nanoparticles and PBz as precursors. By varying the ratio of the precursors (ZnO and PBz), several combination of ZnO@CBs were produced. To determine the photocatalytic activity under UV irradiation, methylene blue (MB) was taken as the investigation molecule. Facile preparation process, low cost, and the uniform hybridization would make the synthesized ZnO@CBs show outstanding ability in the field of sustainable energy and environment.

### Materials

Eugenol, zinc oxide nanoparticles and paraformaldehyde were purchased from Sigma-Aldrich (USA). Ethylene diamine, potassium hydroxide (KOH), sodium hydroxide (NaOH) and dimethyl sulfoxide (DMSO) were purchased from Duksan Chemicals Co., Ltd. Republic of Korea. All chemicals were used without further purification.

## Methods

### Synthesis of benzoxazine monomer (6-allyl-8-methoxy-3-ethylamine-3,4-dihydro-1,3-benzoxazine) (Eu-Bzo)

**Figure 1:**
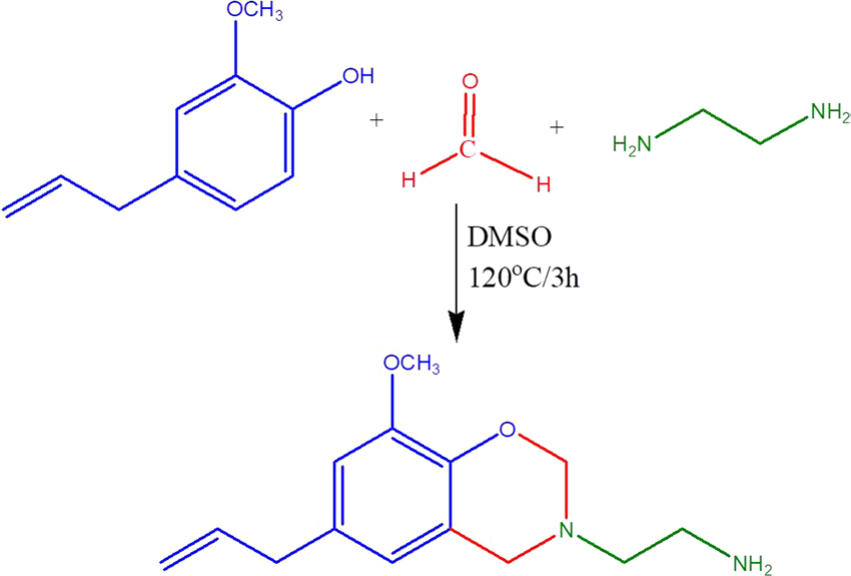
Synthesis of Eu-Bzo.

In a three-necked round-bottomed flask equipped with a magnetic stirrer and a reflux condenser, paraformaldehyde (1.8 g, 0.06 m) was taken and 20 mL of DMSO was added to it. It was then allowed to stir while maintaining the temperature at 50 °C. Ethylenediamine (1.34 mL, 0.02 m) was added dropwise to the stirring mixture. In the meantime, a solution containing eugenol (3.28 g, 0.02 m) in 10 mL of DMSO was prepared separately. After complete addition of ethylenediamine, the solution of eugenol was added dropwise to the reaction mixture. The temperature was then slowly raised to 120 °C. The reaction was then continuously stirred for 3 h at this temperature. On completion of the reaction time, a transparent pale yellow solution was obtained. This solution was then cooled to room temperature and precipitated in a mixture of water and ethanol. The precipitate thus obtained was washed with NaOH solution and distilled water several times, filtered and finally dried in vacuum at 50 °C for 12 h to obtain Eu-Bzo monomer (Fig. 1). Step-wise curing of this Eu-Bzo monomer with the different weight ratios of ZnO (20, 40, 60 and 80%) at 100, 150, 200 and 250 °C, for an hour at each temperature was adopted to produce ZnO/Carbon material. Carbonization of the obtained ZnO/Carbon material was done under nitrogen atmosphere by heating at 700 °C for 5 h with a ramp of 5 °C min^−1^. Activation of the obtained carbonaceous materials was done by thorough mixing in an aqueous KOH solution in a weight ratio of 2:1 (KOH: carbonized material), followed by evaporation at 120 °C to remove the water content. Further, it was activated by heating in a tubular furnace at 700 °C for 1 h. ZnO nanoparticles adorned nitrogen-doped carbon balls thus obtained was denoted as ‘ZnO@CBs’. For comparative studies, carbon balls were prepared by carbonization and activation process in a similar manner from Eu-Bzo monomer without the addition of ZnO, denoted as ‘CBs’. Figure 1 represents the detailed synthesis procedure of Eu-Bzo benzoxazine monomer.

### Photodegradation measurements

The photodegradation measurements of MB dye were carried out in neutral aqueous medium at room temperature under UV-light irradiation by UV-Vis absorbance. The absorbance intensity of approximately 1.5 was fixed for the aqueous MB dye solution. For the typical experiments, 20 mg of the synthesized ZnO@CBs with different wt.% of ZnO/bare ZnO nanoparticles were dropped into 50 mL aqueous MB dye with absorbance intensity of 1.5 and subsequently, the UV-Vis absorbance of aqueous MB dye was measured (0 min) and denoted as blank. This mixture was fixed at a distance of 100 mm from the UV source and then irradiated until the decoloration of aqueous MB dye occurs. During the photodegradation process, the reaction mixture was stirred continuously. The absorbance intensity of aqueous MB dye was measured for every 15 min until the zero (0) intensity of absorbance of aqueous MB dye. The percentage of degradation and degradation rate of MB dye in the presence of ZnO@CBs with different wt.% of ZnO/bare ZnO nanoparticles were calculated and then compared with each other.

## Results and Discussion

### Structure analysis of Eu-Bzo

**Figure 2:**
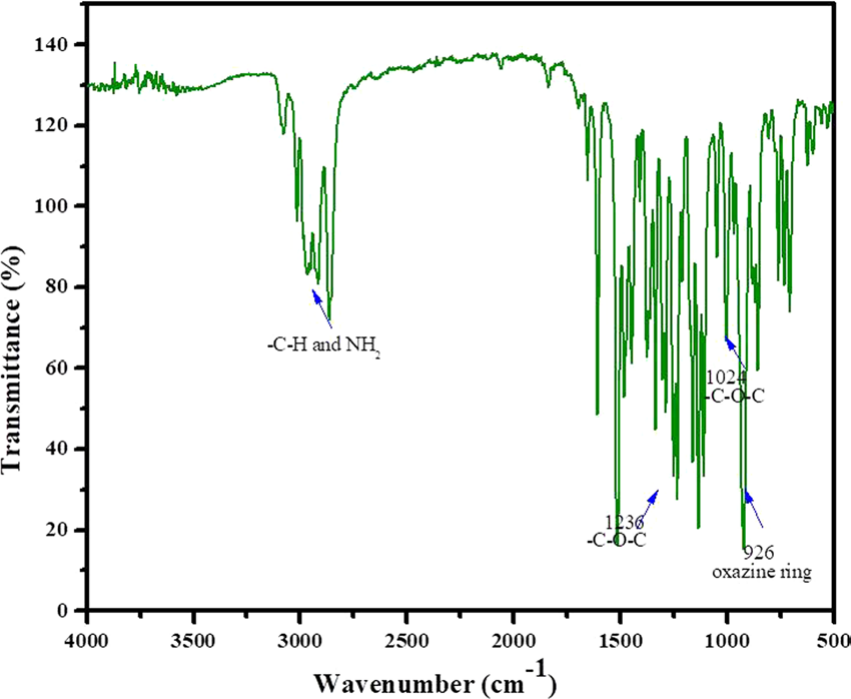
FTIR spectrum of synthesized Eu-Bzo.

The FT-IR spectrum of the benzoxazine monomer was represented in Fig. 2. The band observed at 990 cm^−1^ was due to the –CH_2_ stretching vibration of oxazine ring. The asymmetric and symmetric stretching vibrations of C–O–C and C–N–C were found at 1236 & 1218 cm^−1^ and 1120 & 1094 cm^−1^, respectively (Fig. 2). The stretching vibration of the methoxy group was found at 1272 cm^−1^. The band at 1360 cm^−1^ is due to the tetrasubstituted benzene. Aliphatic C–H stretching vibration was found at 2850 cm^−1^. The stretching vibrations of aromatic –CH and allyl =CH^22–24^ were found between 2906–3076 cm^−1^.

**Figure 3:**
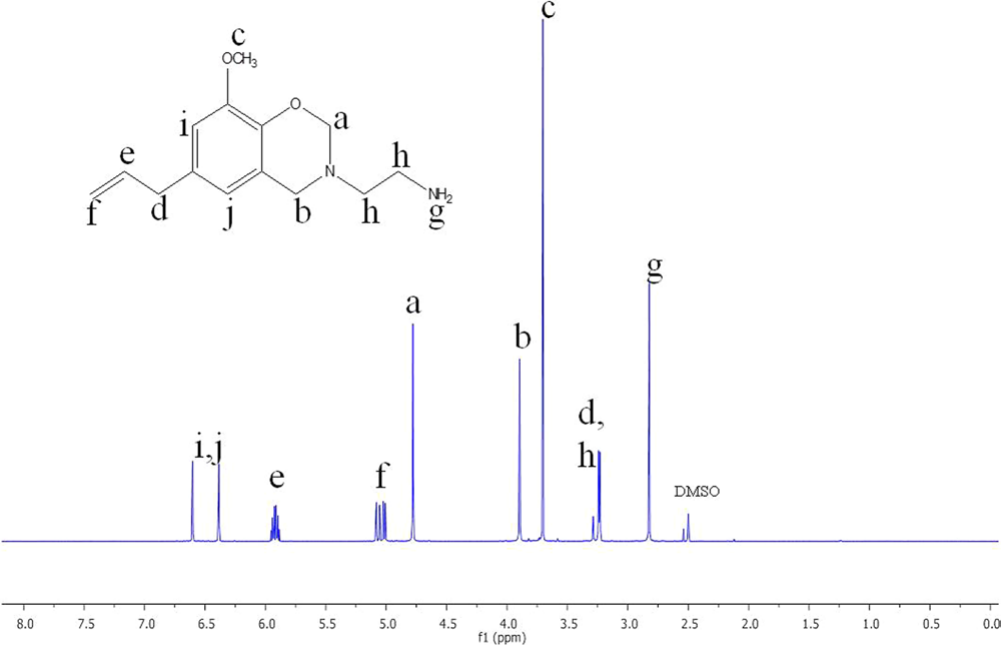
1H-NMR spectrum of synthesized Eu-Bzo.

**Figure 4:**
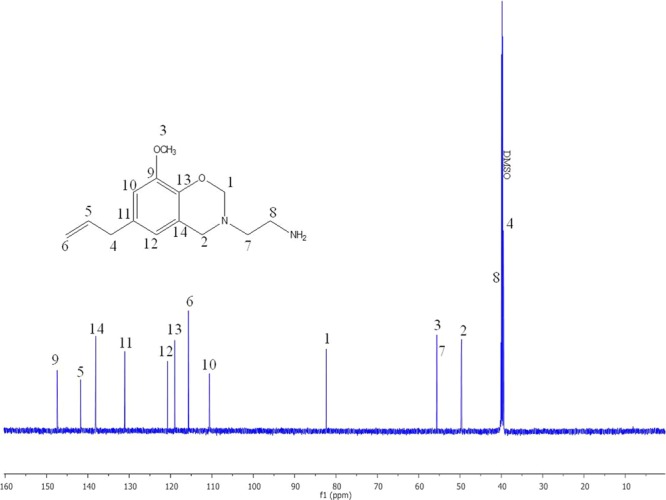
13C-NMR spectrum of synthesized Eu-Bzo.

Figures 3 and 4 represent the ^1^H- and ^13^C-NMR spectra of the benzoxazine monomer. The ^1^H-NMR spectrum shows two singlets at 4.7 and 3.8 ppm due to the oxazine ring protons, O–CH_2_–N and N–CH_2_–Ar, respectively (Fig. 3). The protons of the amine group gave a peak at 2.8 ppm, whereas, the protons of the methoxy groups gave a peak at 3.7 ppm. The methyl protons of ethylamine group resonate at 3.2 ppm and the allyl protons resonate at 5.0 and 5.9 ppm. The aromatic protons gave peaks at 6.3 and 6.6 ppm^25,26^. In the ^13^C–NMR spectrum (Fig. 4), the oxazine ring carbons gave signals at 82.3 ppm for O–C–N and 55.6 ppm for N–C–Ar carbons. Signals at 49.6 and 49.7 ppm were observed for methyl carbons and the allyl carbons resonate at 110–119 ppm. The aromatic carbons resonate between 120–147 ppm. Hence, the structure of the benzoxazine monomer is confirmed by NMR spectroscopic analysis^22,27^.

### Structural analysis of pristine CBs, bare ZnO and ZnO adorned CBs

**Figure 5:**
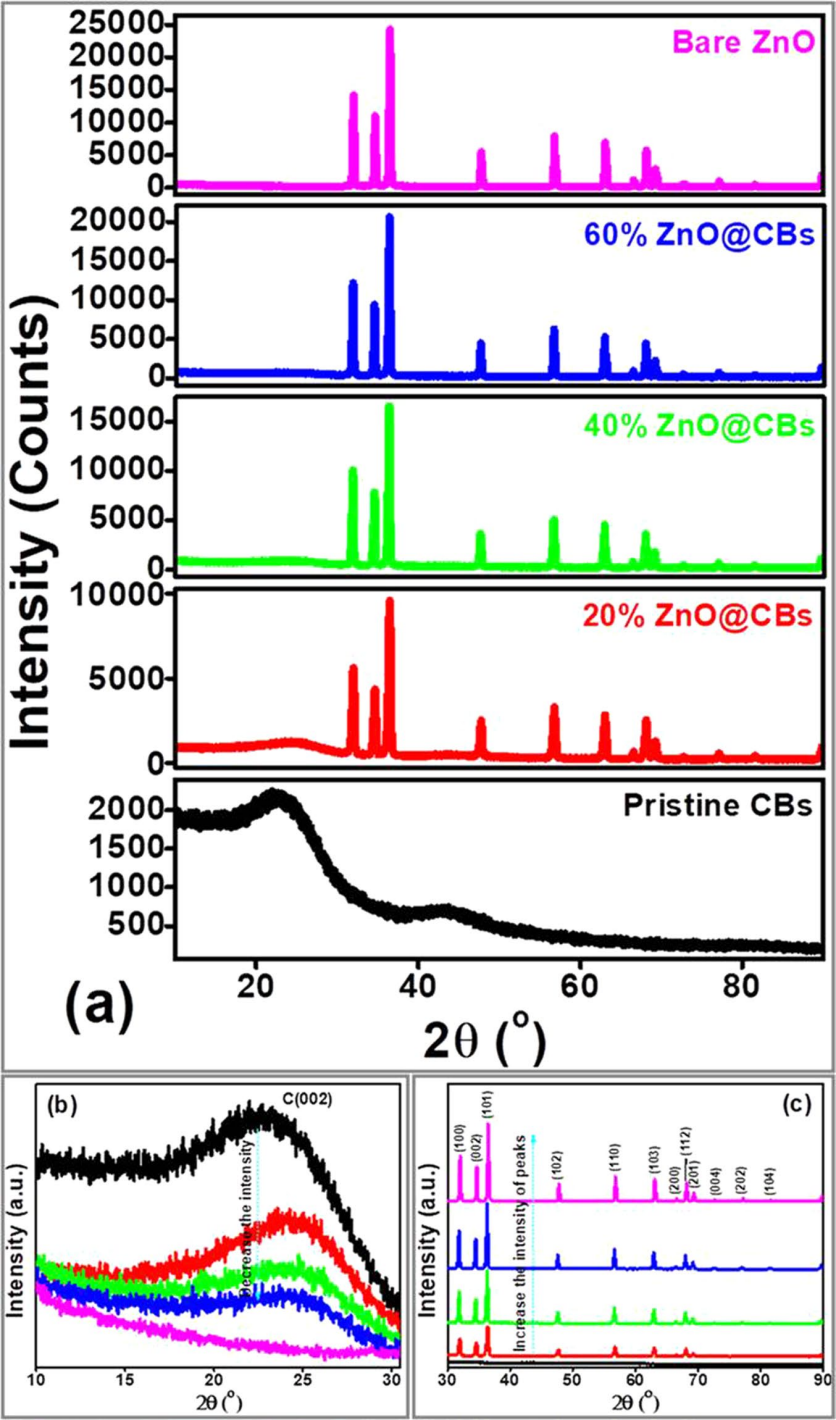
XRD patterns of (**a**) pristine CBs, different wt.% of ZnO@CBs and bare ZnO nanoparticles (**b**) magnified view of carbon peaks and (**c**) magnified view of ZnO peaks.

Crystallinity and graphitization of the synthesized materials were revealed by XRD patterns. Figure 5 shows the typical XRD patterns of the pristine CB, ZnO@CBs with different wt.% of ZnO (20, 40 and 60wt.% of ZnO) and bare ZnO nanoparticles. In the XRD Patterns (Fig. 5a), the pristine CBs exhibits two characteristic diffractions around 23.2 and 43.3° assigned to 002 and 100 planes of typical graphitic carbon^28^. Broad diffraction in the 2θ range between 20–24° was obtained instead of comparatively sharp diffraction for typical graphitic planes (2θ at 26°, JCPDS 41–1487) which might due to the distorted sp^2^ hybrid orbital of carbon atoms and nitrogen atoms bonded carbon in the synthesized pristine CBs. With respect to the ZnO@CB compounds, the diffraction intensities of carbon plane decreased with increasing the amount of the ZnO. This indicates that ZnO nanoparticles are evenly dispersed over the CBs. There are no carbon planes observed in the bare ZnO nanoparticle, as could be seen clearly in Fig. 5b. At the same, the typical diffractions corresponding to graphitic carbon (002 planes) shifts to higher 2θ angle when compared with the pristine CBs. During carbonization, the size of ZnO nanoparticles enlarges within the CBs which compress the carbon structures (reducing the interlayer distance of the corresponding carbon) and resulting in shifting of carbon planes towards higher 2θ values. Moreover, the intensity of typical diffractions corresponding to ZnO in ZnO@CBs increases with increasing the wt.% of ZnO. The bare ZnO presents sharp diffractions with the highest intensity of typical wurtzite hexagonal zinc oxide phase^29–31^, which is clear in Fig. 5c. At the same time, the intensity of ZnO planes enhanced with increasing the wt. % of ZnO. Indeed, the interaction of ZnO and also the ionic radius of C (0.260 nm) is much larger than that of O_2_ (0.140 nm). Thus, the interaction of O (ZnO) with C (carbon ball) during the high-temperature calcination essentially enlarges the lattice of ZnO, affecting a peak shift toward higher angles for the CBs component of ZnO@CBs, which proposes the possible C-doping of ZnO in the resultant ZnO@CBs compounds, XRD patterns (Fig. 5c), the typical diffractions corresponding to ZnO for ZnO@CBs the diffraction intensities of ZnO increases with increasing of the ZnO while pure ZnO presents the sharp diffractions of typical Wurtzite hexagonal zinc oxide phase^30^.

**Figure 6:**
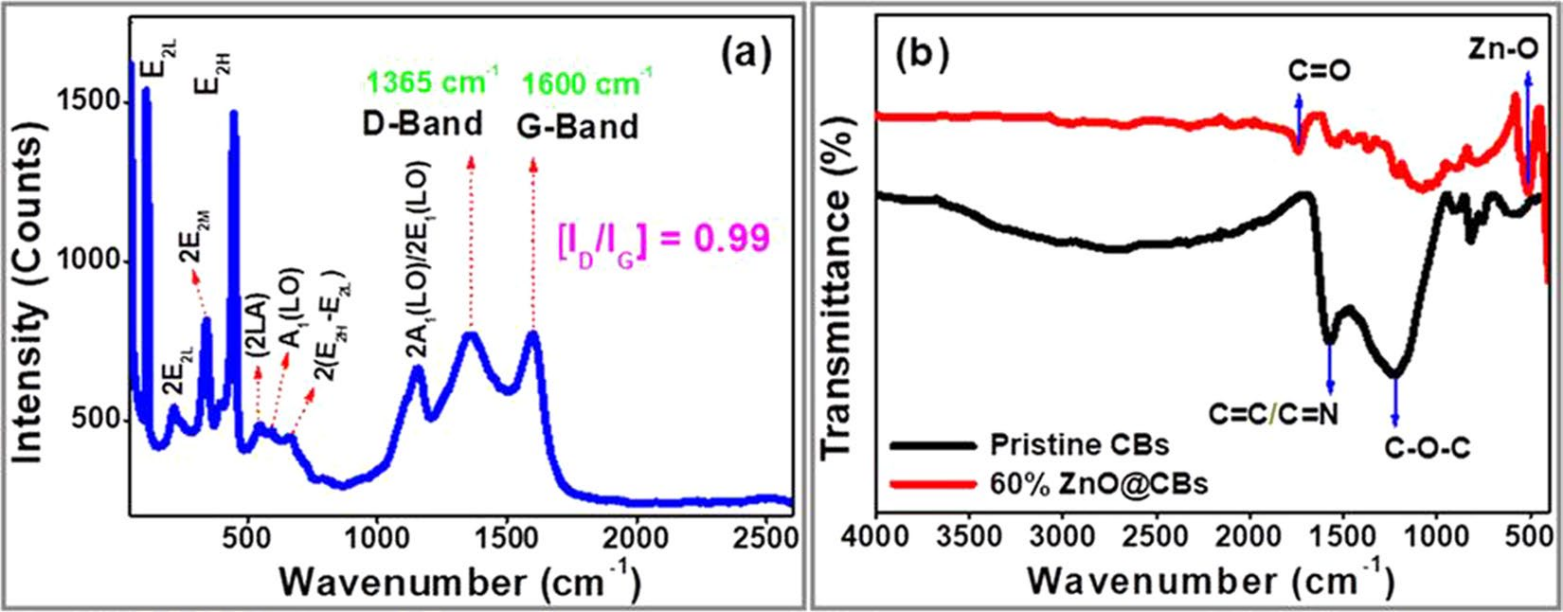
(**a**) Raman spectrum of 60 wt.% ZnO@CBs and (**b**) FTIR spectra of pristine CBs and 60 wt.% ZnO@CBs.

Raman Spectroscopy technique is an important and very sensitive method to examine the ordered graphitic/crystal structures of the carbon materials. Figure 6a shows the Raman spectrum of the synthesized ZnO@CBs (60 wt.%). As shown in the Raman spectrum, the carbon materials in the ZnO@CBs gave G-band at 1601 cm^−1^ which is typical of sp^2^ hybridized carbon (E_2g_) and the D-band at 1360 cm^−1^ which is typical of sp^3^ hybridized carbon (A_1g_)^32^. Raman intensity ratio (I_D_/I_G_) is a measurement of the degree of graphitization in the ZnO@CBs. The I_D_/I_G_ is 0.99 in this sample, indicating that the carbons have adequate graphitic structure^33^. In addition, the bands exhibited at 1158, 667, 593, 547, 447, 343, 218, and 111 cm^−1^ are attributed to 2A_1_(LO)/2E_1_(LO), 2(E_2H_-E_2L_), A_1_(LO), 2LA, E_2H_, 2E_2M_, 2E_2L_, and E_2L_ modes of the ZnO Wurtzite structure. This result indicates the ZnO nanoparticles present over the CBs in the ZnO@CBs. FT-IR spectroscopy is used to determine the functional groups in the synthesized pristine CB and ZnO@CBs which are shown in Fig. 6b. In the spectrum of pristine CBs, the characteristic band of C=C aromatic stretching vibration at 1592 cm^−1^ indicates the presence of sp^2^ hybridized honeycomb lattice^34^. The aromatic heterocyclic C=N stretching vibrations at 1560 cm^−1^ is merged with C=C aromatic stretching vibration that can be observed clearly in the spectrum. The oxygen-containing functional groups such as C–OH and C–O–C stretching vibration^32–34^ on the surface of the carbon structure appeared between 1350–1050 cm^−1^. The FT-IR spectrum of ZnO@CBs (60 wt.%) is similar to the pristine CBs in the characteristic bands suggesting the characteristic graphitic arrangement of CBs was well reserved after uniform mixing with ZnO. However, the typical band intensity of graphitic CBs is weakened suggesting the conjugated structures of CBs are strained due to the addition of ZnO nanoparticles^35,36^. In addition, C=O and Zn–O stretching vibrations appeared at 1740 and 514 cm^−1^, respectively. The appearance of C=O groups might be due to disorder with the oxidation of the surface of the CBs by the addition of ZnO nanoparticles. The result suggests that the absence of a chemical bond between the C and Zn due to the absence of C–Zn stretching vibrations.

**Figure 7:**
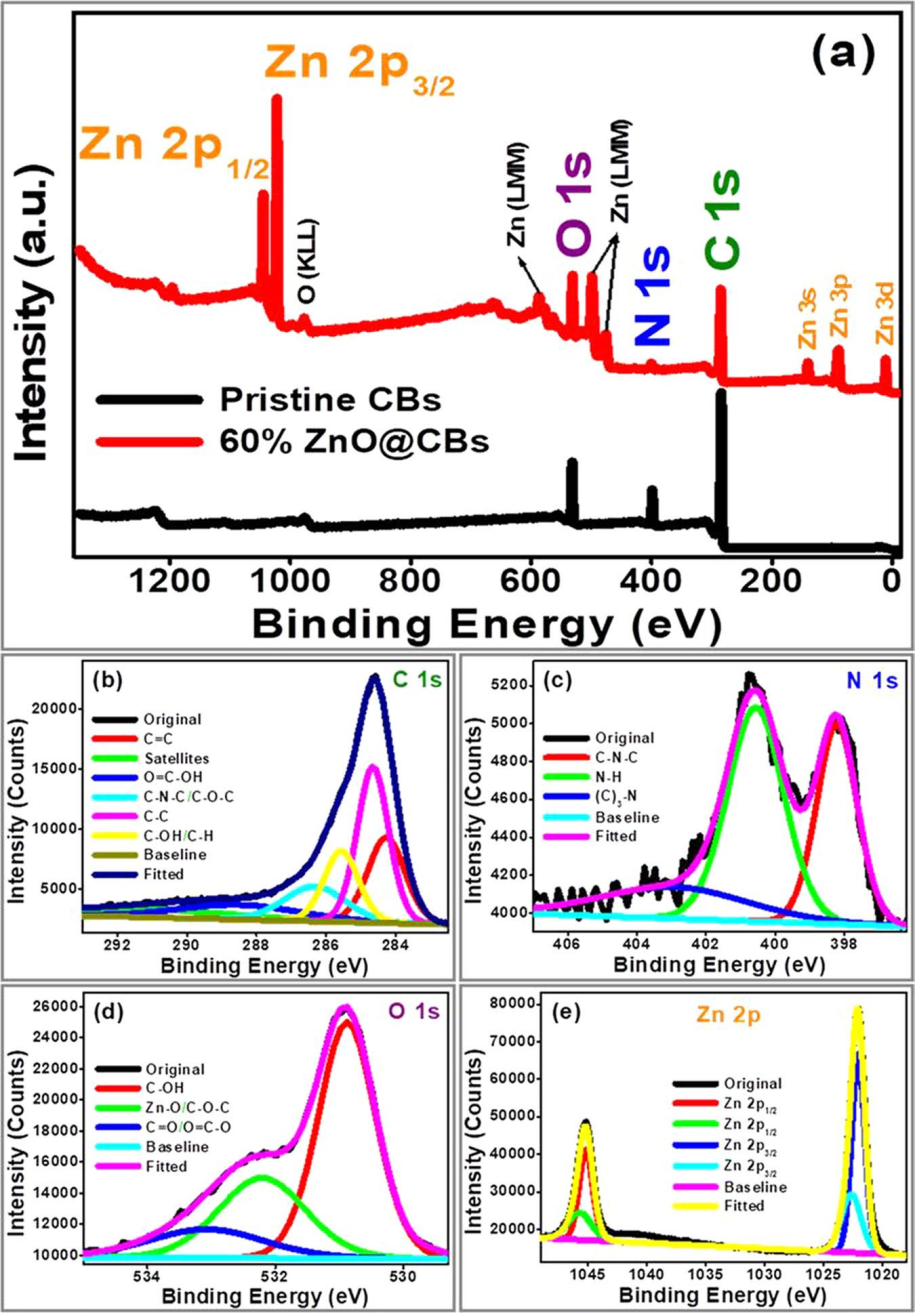
(**a)** XPS survey spectra of pristine CBs and 60 wt.% ZnO@CBs (**b**) C 1s spectrum (**c**) N 1s spectrum (**d**) O 1s spectrum and (**e**) Zn 2p spectrum of 60 wt.% ZnO@CBs.

Further, the functionalities and elemental composition of the synthesized pristine CBs and ZnO@CBs were confirmed by XPS. Figure 7 shows the XPS of pristine CBs and ZnO@CBs (60 wt.%). In the survey spectrum of pristine CBs three distinct peaks at the binding energy of 284.1, 399.1 and 532.1 eV correspond to the C 1s, N 1s and O 1s energy levels, respectively (Fig. 7a). Whereas, the survey spectrum of ZnO@CBs displays the C 1s, N 1s and O 1s energy levels with the addition of Zn energy peaks, mainly the Zn 2p energy levels at 1022.1 and 1045.1 eV. The high-resolution XPS of C1 s, N 1s, O 1s and Zn 2p energy levels for ZnO@CBs were measured and deconvoluted using casaXPS. The C 1s spectrum can be de-convoluted into five components at 284.3, 284.7, 285.6, 286.4 and 288.7 eV, assigned to hydrocarbon chains of the (C=C), hydrocarbon chains (C–C)/carbon atoms in C–N bond, C–OH/C–H, C–N–C/C–O–C groups and HO–C=O group carbon atom bonded with oxazine, respectively (Fig. 7b). The peak positioned at 284.7 eV is assigned to pure graphitic sites in the carbon family; however, the energy influence at 285.6 eV is ascribed to the sp^2^-hybridized C–N bonding in an aromatic ring^16,37^. The peak positioning at 288.7 eV is due to the sp^2^-hybridized carbon in the aromatic ring attached to the nitrogen atom. The high-resolution N 1s XPS can be deconvoluted into two main peaks and one weak peak, the first main peak at 398.2 eV is attributed to the aromatic N, bonded to two carbon atoms (C–N–C), and second main peak at 400.6 eV corresponds to N, bonded to the hydrogen atom (N–H) (Fig. 7c). The weak peak at 402.7 eV corresponds to the sp^2^-hybridized N, bonded to three atoms [N–(C)_3_]^38–40^. The high-resolution O 1s XPS can be deconvoluted into three peaks, the main peak centered at 530.9 eV is assigned to C–OH, the peak positioned at 532.2 eV is allocated to O^2−^ ions in the Zn–O bonding of the Wurtzite ZnO structure, and the weak peak located at 533.0 eV is associated to C=O/O=C–OH/OH groups absorbed onto the surface of the compound (Fig. 7d). The Zn 2p spectrum shows two binding energy of 1022.1 and 1045.2 eV, assigned to Zn 2p_3/2_ and 2p_1/2_ lines, respectively^41^. The binding energy gap between these two peaks is 23.1 eV, this value matches with the reference value of ZnO (Fig. 7e). There is no peak corresponding to C–Zn bond in the XPS spectrum, which confirms the absence of chemical bond between C and Zn. Hence, the ZnO nanoparticles are physically adsorbed over the CBs, which is similar to the results observed from FTIR.

**Figure 8:**
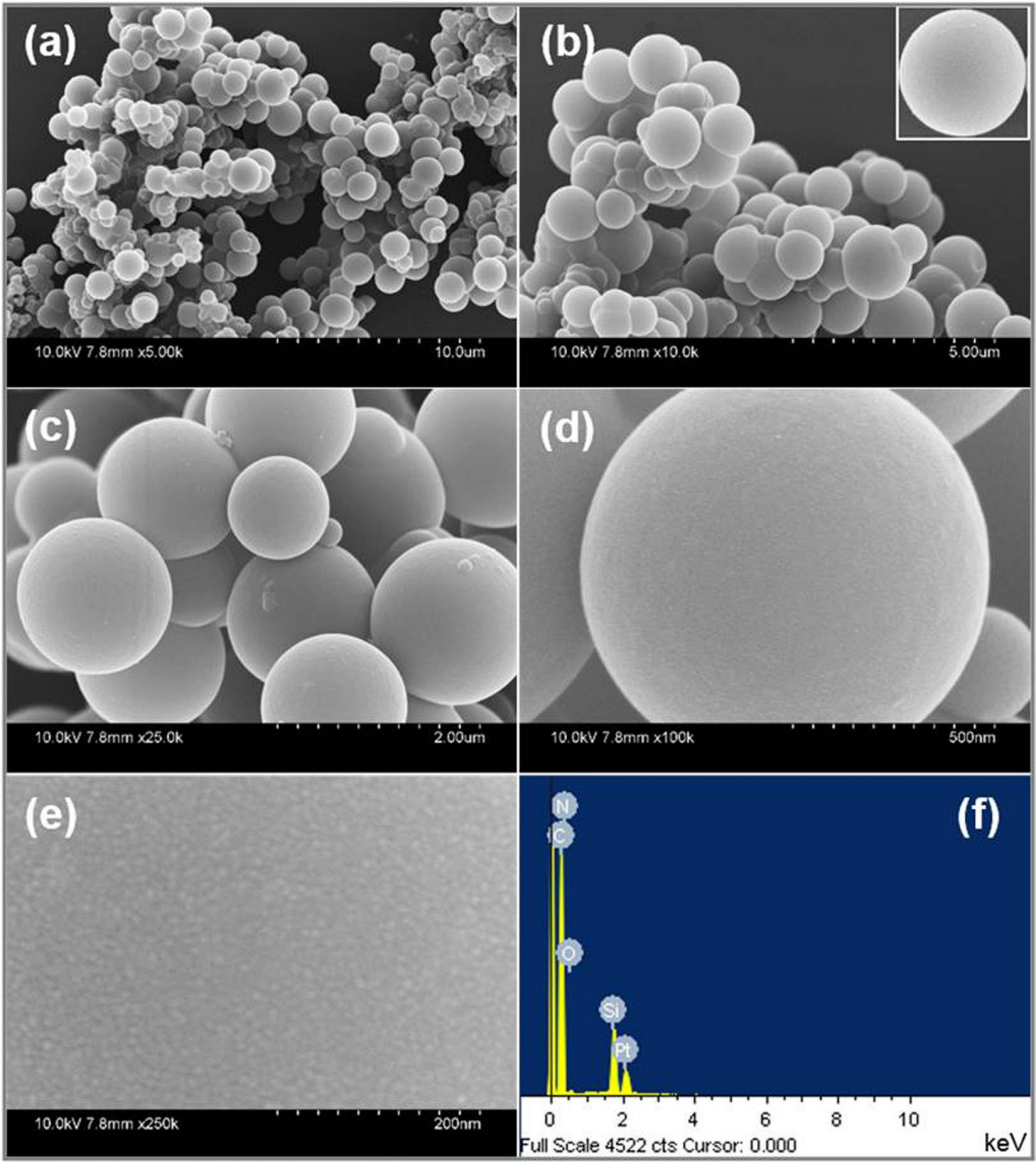
(**a–e**) FESEM images of pristine CBs with different magnifications and (**f**) EDX spectrum of pristine CBs.

**Figure 9:**
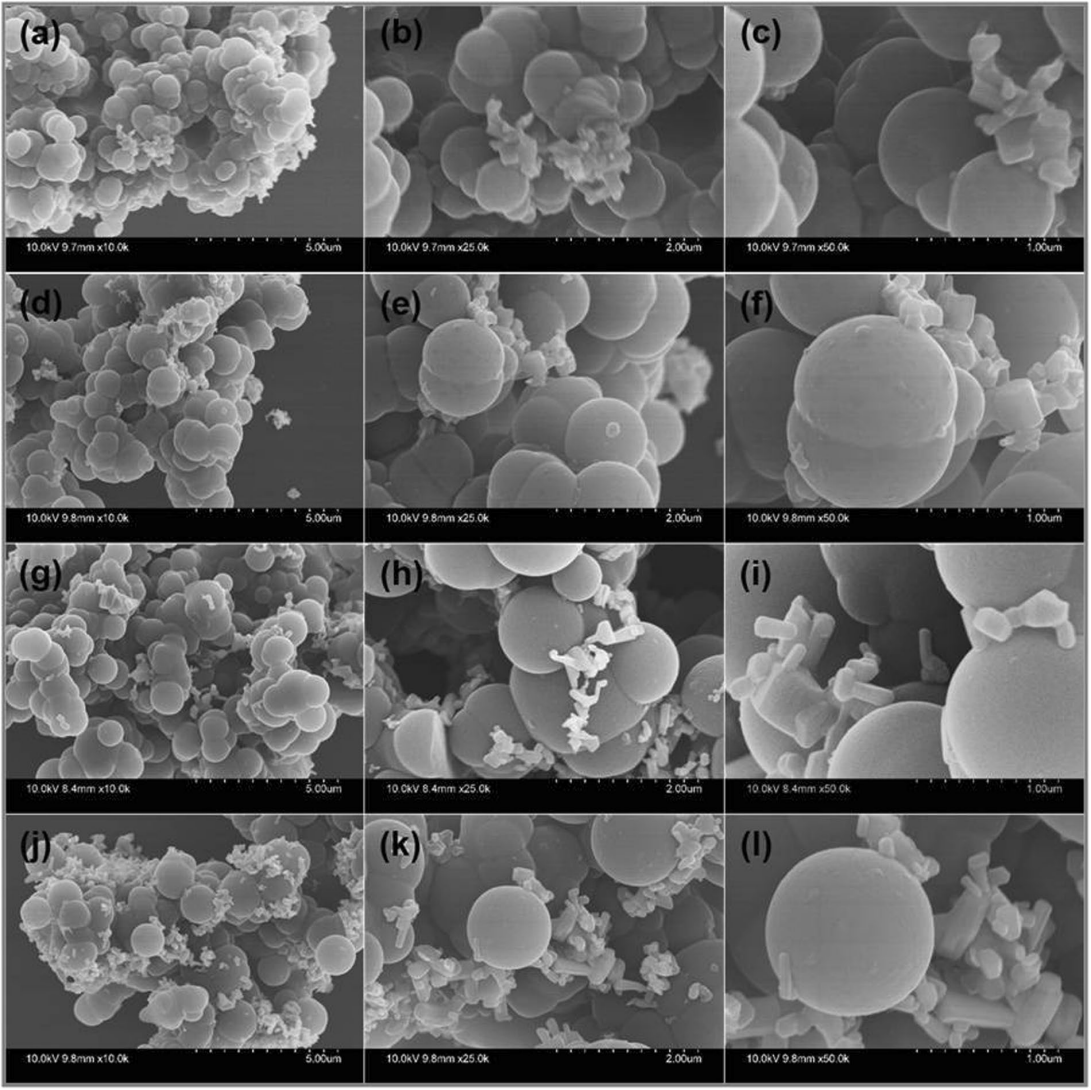
FESEM images of ZnO@CBs with different magnifications (**a–c**) 20 wt.% (**d–f**) 40 wt.% (**g–i**) 60 wt.% and (**j–l**) 80 wt.%.

**Figure 10:**
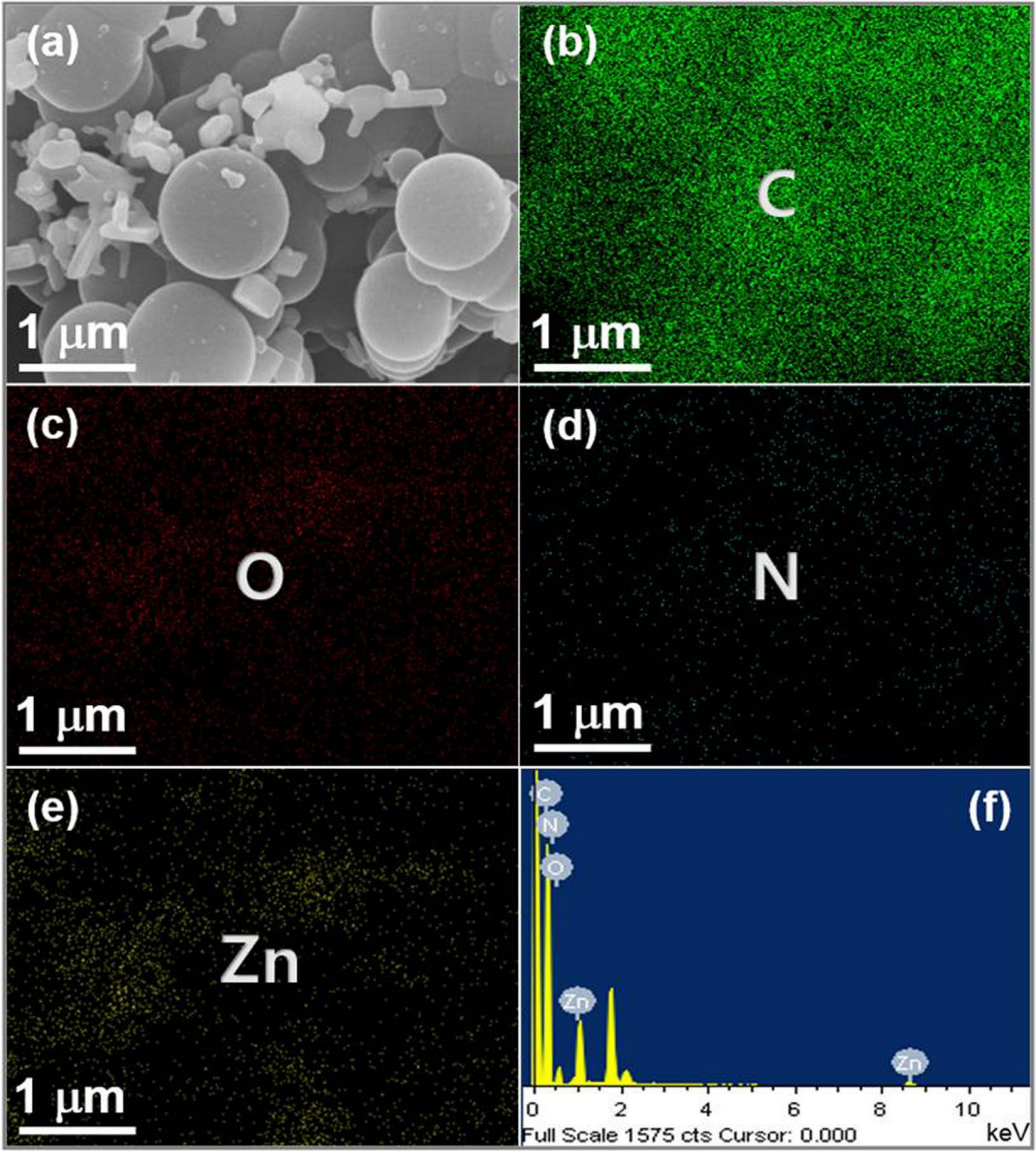
(**a**) FESEM image and (**b–e**) the corresponding elemental mapping and (**f**) EDX spectrum of 60 wt.% ZnO@CBs.

The surface morphology of the synthesized pristine CBs and ZnO@CBs were examined through FESEM analysis. The FESEM images of pristine CBs at different magnifications are shown in Fig. 8(a–e). The particles are ball-like and mono-dispersed with smooth morphology as could be seen from the images. The high magnification FESEM image exhibits that the prepared CBs has porous structure that is made by the construction of numerous nanoparticles resulting in micro-porous surface. The average diameter of the pristine CBs is around 500–1000 nm. The elemental composition of the synthesized pristine CBs is supported by the EDX spectrum (Fig. 8f). In the EDX spectrum, the constituting elements C, N, and O clearly display a well-defined compositional profile of the hybrid with an atomic percentage of 60.8, 12.6 and 26.6, respectively. Figure 9(a–l) shows the FESEM images of different wt.% (20, 40, 60 and 80 wt. %) of ZnO loaded CBs (ZnO@CBs). All ratios of ZnO@CBs are composed of sponge-like balls with ZnO nanoparticles. With increasing amount of ZnO nanoparticles, more densely packed ZnO nanoparticles on CBs could be found. The CBs in the ZnO@CBs also seems to be like pristine CBs, but it exhibits little aggregation compared to pristine CBs. This could be owing to the effect of the water released from the precursor during ring-opening polymerization followed by decomposition and condensation at high-temperature calcination in the argon atmosphere^42,43^. This well-structured porosity may be favorable for the photocatalysis due to the enhancement of mass transfer through the materials. Moreover, the ZnO nanoparticles are uniformly distributed over the CBs, as confirmed by elemental mapping of ZnO@CBs. Elemental analysis was accomplished to approve the presence of elements in the synthesized ZnO@CBs. Figure 10 shows the elemental mapping and EDX spectrum of 60 wt.% ZnO@CBs. Elemental mapping (Fig. 10(b–e)) shows the presence of the elements (C, O, N, and Zn) in the resulted ZnO@CBs. In addition, N and Zn are evenly distributed within the carbon structure (CBs). The elemental composition of 60 wt.% ZnO@CBs was further revealed through the EDX spectrum (Fig. 10f), in which only C, N, O and Zn signals were identified. In addition, the peak around 1.8 keV corresponds to Si which originates from the silicon wafer substrate (sample was dispersed on the silicon wafer for the FESEM with EDX analysis). Apart from that, no other signal of subordinate phase or contamination was detected in the EDX spectrum. This designates the high purity of the chemical compound (ZnO@CBs) that matches well with the XPS result.

**Figure 11:**
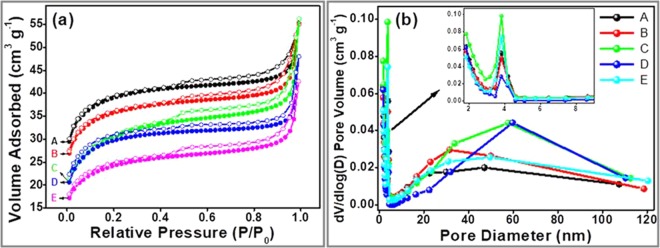
Surface area measurement (**a**) nitrogen adsorption-desorption isotherms and (**b**) corresponding pore size distribution of different wt.% of ZnO@CBs (A) pristine CBs (B) 20 wt.% ZnO@CBs (C) 40 wt.% ZnO@CBs (D) 60 wt.% ZnO@CBs (E) 80 wt.% ZnO@CBs.

The specific surface area and the pore size of the synthesized ZnO@CBs were investigated by nitrogen adsorption-desorption isotherms as shown in Fig. 11. The synthesized ZnO@CBs with different wt.% (Fig. 11a) exhibit a type IV isotherm with H_3_ hysteresis loop, the sharp capillary condensation step at high relative pressures (P/P0) >0.9, demonstrating the existence of large pores in the CBs. These pores found to have both mesoporous and macroporous structure, but with enormous amount of mesopores^44,45^. The Brunauer–Emmet–Teller (BET) surface area of pristine CBs, 20 wt.% ZnO@CBs, 40 wt.% ZnO@CBs, 60 wt.% ZnO@CBs and 80 wt.% ZnO@CBs was measured as 290, 250, 232, 217 and 180 m^2^ g^−1^, respectively. The surface area of ZnO@CBs gradually decreased with increasing the concentration of ZnO from 20 to 80 wt.%, as some of the pores in the CBs were blocked/occupied by the ZnO nanoparticles. Figure 11b shows the pore size distribution graph of the synthesized 20 wt.% ZnO@CBs, 40 wt.% ZnO@CBs, 60 wt.% ZnO@CBs and 80 wt.% ZnO@CBs composites, the graphs display the existence of mesopores and macropores with all percentages of ZnO@CBs. The result from BET surface area concludes that adequate space is available in CBs (ZnO@CBs) for moving aqueous dyes from one side to another without any struggle. ZnO@CBs with high surface area and large pore size will make a positive contribution to its photocatalytic performance.

### Photocatalytic performance of the bare ZnO and ZnO adorned CBs

**Figure 12:**
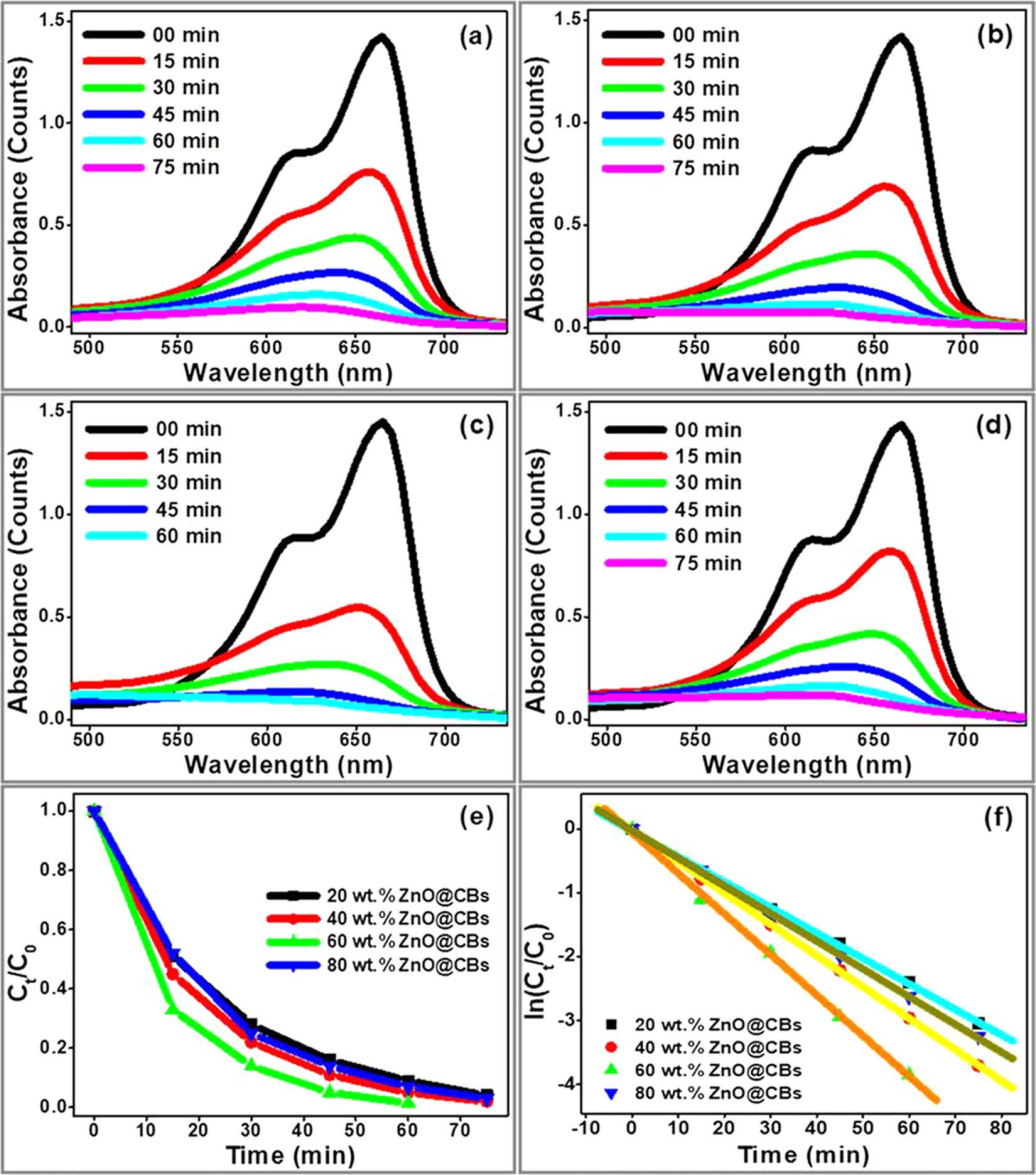
Degradation of MB dye in presence of (**a**) 20 wt.% ZnO@CBs (**b**) 40 wt.% ZnO@CBs (**c**) 60 wt.% ZnO@CBs and (**d**) 80 wt.% ZnO@CBs at different time intervals (min) under UV light (**e**) UV light photocatalytic performance of the different wt. % of ZnO@CBs in degradation of MB and (**f**) Reaction kinetics of degradation of MB over different wt.% of ZnO@CBs.

In order to determine the efficiency of the bare ZnO and synthesized ZnO adorned CBs on the degradation of MB dye under UV light, the UV–Vis absorption spectra were used at different irradiation time. Figure 12 displays the UV–Vis absorption spectra of the MB solution in the presence of synthesized photocatalysts (different wt.% of ZnO@CBs) under UV light illumination for different time intervals. Figure 12a–d corresponds to the UV–Vis absorption spectra of the MB solution in the presence of photocatalyst, i.e., 20 wt.% of ZnO@CBs, 40 wt.% of ZnO@CBs, 60 wt.% of ZnO@CBs and 80 wt.% of ZnO@CBs. As shown in Fig. 12a–d, the absorption intensity decreases with increasing the irradiation time, indicating a progressive degradation of MB by the destruction of its aromatic ring, and this decrement follows different trends for each photocatalyst. Similarly, the decrease of the MB absorbance in the presence of bare ZnO nanoparticles at irradiation time under UV light is shown in Fig. S1. The MB dye degradation percentages for 20 wt.% of ZnO@CBs, 40 wt.% of ZnO@CBs, 60 wt.% of ZnO@CBs, 80 wt.% of ZnO@CBs and bare ZnO nanoparticles were found to be 92, 96, 99, 94, and 87%, respectively after 60 min of UV light irradiation. ZnO@CBs with 60 wt.% loading of ZnO showed enhanced photocatalytic activity when compared with other wt.% of ZnO@CBs photocatalyst (Fig. 12e). The degradation rates were 1.53% min^−1^ (rate constant (k) = 0.0399 min^−1^; R^2^ = 0.999), 1.6% min^−1^ (rate constant (k) = 0.0491 min^−1^; R^2^ = 0.999), 1.65% min^−1^ (rate constant (k) = 0.0636 min^−1^; R^2^ = 0.999), 1.57% min^−1^ (rate constant (k) = 0.0434 min^−1^; R^2^ = 0.999), and 1.45% min^−1^ (rate constant (k) = 0.0319 min^−1^; R^2^ = 0.998), respectively for 20 wt.% of ZnO@CBs, 40 wt. % of ZnO@CBs, 60 wt. % of ZnO@CBs, 80 wt. % of ZnO@CBs and bare ZnO nanoparticles under the UV light irradiation (Fig. 12f). The CBs assisted ZnO nanoparticles (ZnO@CBs) delivered a good photocatalytic performance towards the degradation of MB dye compared to the bare ZnO nanoparticles which are due to the synergetic effect between the carbon and ZnO. Among the ZnO@CBs, 60 wt.% of ZnO@CBs shows an excellent photodegradation performance due to the optimum amount of photocatalyst (ZnO nanoparticles) and also the acceptable surface area. The photocatalytic performance of ZnO@CBs increases with increasing the amount of ZnO nanoparticle up to 60 wt.% and beyond this wt.%, it decreased. The photocatalytic performance towards the degradation of MB dye is as follows: 60 wt.% of ZnO@CBs > 40 wt.% of ZnO@CBs > 80 wt.% of ZnO@CBs > 20 wt.% of ZnO@CBs > bare ZnO nanoparticles. In general, the photocatalytic activity is not only correlated with electron transportation of photocatalysts but also depends on the surface area and amount of active nanoparticles. Hence, the photocatalytic performance of 60 wt.% of ZnO@CBs is higher than that of the other synthesized photocatalyst. The nitrogen adsorption-desorption isotherms strongly supports the above discussion.

### Degradation mechanism of MB dye using ZnO@CBs under UV light illumination

**Figure 13:**
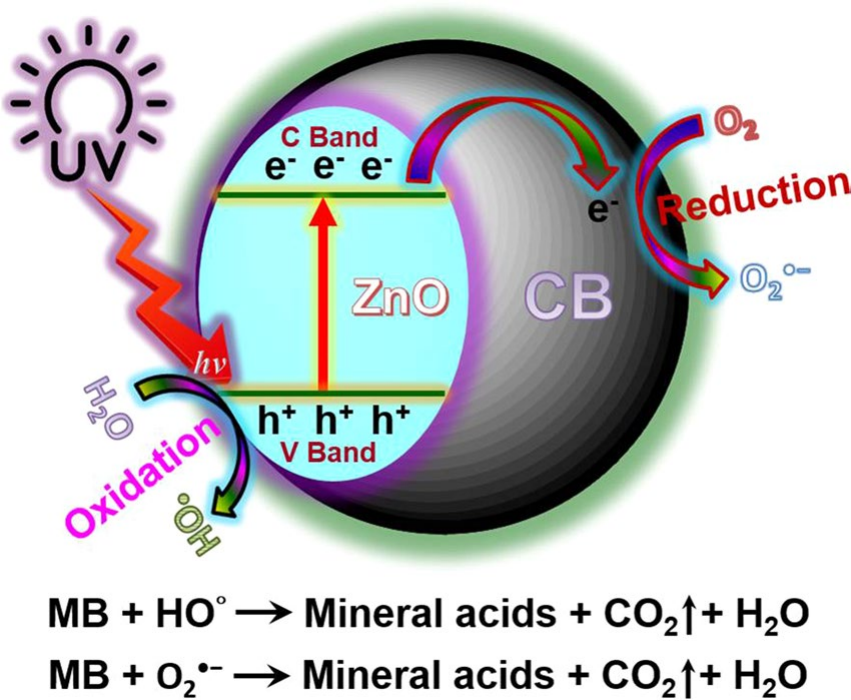
Plausible degradation mechanism of MB dye using ZnO@CBs photocatalyst under UV light illumination.

Photocatalytic activity of the synthesized ZnO@CBs (towards degradation of MB in the aqueous solution under UV-light irradiation) and photocatalytic mechanism have been proposed on the basis of previous reports^46–48^. Electrons get excited and are promoted from its valence band (V band) to conduction band (C band), when ZnO was irradiated under the UV light. The positive holes (h^+^) and negative-electron (e^−^) were generated in the V band and C band of ZnO, respectively under the UV light irradiation. The energy difference between the V band and the C band is denoted as the band-gap. The negative electrons in the C band of ZnO are easily transferred to C band of CBs under the UV light irradiation. Irradiation with UV light leads to the excitation of MB dye molecules adsorbed onto the ZnO@CBs. Subsequently, the ZnO and CBs within ZnO@CBs might involve in the photocatalytic reaction under the UV light. The photogenerated electrons (e^−^) created by the above mentioned processes react with dissolved O_2_ molecules forming superoxide anion radicals (O_2_^•−^), while holes (h^+^) react with H_2_O leading to the formation of hydroxyl radicals (^•^OH) that undergoes chain reactions. Both of the photodegradation processes cause the degradation of the MB dye and are schematically illustrated in Fig. 13.

## Conclusions

In summary, a novel ZnO@CBs photocatalyst was successfully synthesized using Eu-Bzo as the source of carbon and ZnO nanoparticles through a simple carbonization method. The synthesized ZnO@CBs exhibited good crystallinity/graphitization, as revealed by XRD and Raman spectroscopy; uniform size distribution, as depicted by FESEM; photocatalytic performance on MB dye, as depicted by UV-vis spectral measurement. ZnO@CBs displayed enhanced photocatalytic activity compared to the bare ZnO nanoparticles, indicating the synergistic phenomenon between CBs and the semiconducting ZnO. The maximum degradation efficiency of 99.9% with the degradation rate of 1.65% min^−1^ was achieved after 60 min of UV light irradiation. The results suggest that the synthesized ZnO@CBs photocatalyst is a potential and effective material for extensive pollutant removal. Moreover, the proposed technique may be used for the synthesis of numerous metal oxide adorned CB composite materials using Eu-Bzo and addressing the current issue of environmental pollution caused by various organic pollutants.

## Supplementary information

Supplementary Information

## References

[1] Adeleye AS (2016). Engineered nanomaterials for water treatment and remediation: Costs, benefits and applicability. Chem. Eng. J..

[2] Padaki M (2015). Membrane technology enhancement in oil-water separation. A review. Desalination.

[3] Ahmad A (2015). Recent advances in new generation dye removal technologies: novel search for approaches to reprocess wastewater. RSC Adv..

[4] Ahn KS (2008). ZnO nanocoral structures for photoelectrochemical cells. Appl. Phys. Lett..

[5] Yang X (2009). Nitrogen-doped ZnO nanowire arrays for photoelectrochemical water splitting. Nano Lett..

[6] Kochuveedu ST, Jang YH, Jang YJ, Kim DH (2013). Visible light active photocatalysis on block copolymer induced strings of ZnO nanoparticles doped with carbon. J. Mater. Chem. A.

[7] Matos J, Garcia A, Zhao L, Titirici MM (2010). Solvothermal carbon-doped TiO_2_ photocatalyst for the enhanced methylene blue degradation under visible light. Appl. Catal. A.

[8] Zhu Y-P (2014). Highly dispersed photoactive zinc oxide nanoparticles on mesoporous phosphonated titania hybrid. Appl. Catal. B.

[9] Qin H, Li W, Xia Y, He T (2011). Photocatalytic activity of heterostructures based on ZnO and N-doped ZnO. ACS Appl. Mater. Interfaces.

[10] Qu K (2017). Promotion of electrocatalytic hydrogen evolution reaction on nitrogen-doped carbon nanosheets with secondary heteroatoms. ACS Nano.

[11] Jia S (2016). An efficient preparation of N-doped mesoporous carbon derived from milk powder for supercapacitors and fuel cells. Electrochim. Acta.

[12] Zhu G (2017). Pine needle-derived microporous nitrogen-doped carbon frameworks exhibit high performances in electrocatalytic hydrogen evolution reaction and supercapacitors. Nanoscale.

[13] Zuo S (2018). Preparation of 3D interconnected hierarchical porous N-doped carbon nanotubes. Carbon.

[14] Davodi F, Tavakkoli M, Lahtinen J, Kallio T (2017). Straightforward synthesis of nitrogen-doped carbon nanotubes as highly active bifunctional electrocatalysts for full water splitting. J. Catal..

[15] Wan L, Wang J, Xie L, Sun Y, Li K (2014). Nitrogen-enriched hierarchically porous carbons prepared from polybenzoxazine for high-performance supercapacitors. ACS Appl. Mater. Interfaces.

[16] Wang S (2014). Diaminohexane-assisted preparation of coral-like, poly(benzoxazine)-based porous carbons for electrochemical energy storage. ACS Appl. Mater. Interfaces.

[17] Wan L, Wang J, Sun Y, Feng C, Li K (2015). Polybenzoxazine-based nitrogen-containing porous carbons for high-performance supercapacitor electrodes and carbon dioxide capture. RSC Adv..

[18] Wang S, Li W-C, Zhang L, Jin Z-Y, Lu A-H (2014). Polybenzoxazine-based monodisperse carbon spheres with low-thermal shrinkage and their CO_2_ adsorption properties. J. Mater. Chem. A.

[19] Thubsuang U, Ishida H, Wongkasemjit S, Chaisuwan T (2015). Advanced and economical ambient drying method for controlled mesopore polybenzoxazine-based carbon xerogels: Effects of non-ionic and cationic surfactant on porous structure. J. Colloid Interface Sci..

[20] Thubsuang U, Sukanan D, Sahasithiwat S, Wongkasemjit S, Chaisuwan T (2015). Highly sensitive room temperature organic vapor sensor based on polybenzoxazine-derived carbon aerogel thin film composite. Mater. Sci. Eng. B.

[21] Thirukumaran P, Shakila A, Sarojadevi M (2014). Synthesis and characterization of novel bio-based benzoxazines from eugenol. RSC Adv..

[22] Wang C, Sun J, Liu X, Sudo A, Endo T (2012). Synthesis and copolymerization of fully bio-based benzoxazinesfrom guaiacol, furfurylamine and stearylamine. Green Chem..

[23] Macko JA, Ishida H (2001). Structural effects of phenols on the photooxidative degradation of polybenzoxazines. Polymer.

[24] Thirukumaran P, Shakila Parveen A, Sarojadevi M (2014). Synthesis and copolymerization of fully biobased benzoxazines from renewable resources. ACS Sustainable Chem. Eng..

[25] Shen X, Dai J, Liu Y, Liu X, Zhu J (2017). Synthesis of high performance polybenzoxazine networks from bio-based furfurylamine: Furan *vs* benzene ring. Polymer.

[26] Thirukumaran P, Shakila Parveen A, Sarojadevi M (2015). New benzoxazines containing polyhedral oligomeric silsesquioxane from eugenol, guaiacol and vanillin. New J. Chem..

[27] Agag T, Arza CR, Maurer FHJ, Ishida H (2010). Primary amine-functional benzoxazine monomers and their use for amide-containing monomeric benzoxazines. Macromolecules.

[28] Atchudan R, Perumal S, Edison TNJI, Lee YR (2016). Facile synthesis of monodisperse hollow carbon nanospheres using sucrose by carbonization route. Mater. Lett..

[29] Zhu Y-P, Li M, Liu Y-L, Ren T-Z, Yuan Z-Y (2014). Carbon-doped ZnO hybridized homogeneously with graphitic carbon nitride nanocomposites for photocatalysis. J. Phys. Chem. C.

[30] Portillo-Velez NS, Hernandez-Gordillo A, Bizarro M (2017). Morphological effect of ZnO nanoflakes and nanobars on the photocatalytic dye degradation. Catal. Today.

[31] Pan L (2015). TiO_2_–ZnO composite sphere decorated with ZnO clusters for effective charge isolation in photocatalysis. Ind. Eng. Chem. Res..

[32] Atchudan R, Edison TNJI, Perumal S, Karthikeyan D, Lee YR (2016). Facile synthesis of zinc oxide nanoparticles decorated graphene oxide composite via simple solvothermal route and their photocatalytic activity on methylene blue degradation. J. Photochem. Photobiol. B.

[33] Atchudan R (2019). An ultrasensitive photoelectrochemical biosensor for glucose based on bio-derived nitrogen-doped carbon sheets wrapped titanium dioxide nanoparticles. Biosens. Bioelectron..

[34] Zhao J (2018). Autocatalysis synthesis of poly (benzoxazine-*co*-resol)-based polymer and carbon spheres. Macromolecules.

[35] Wang X (2016). Rapid construction of ZnO@ZIF-8 heterostructures with size-selective photocatalysis properties. ACS Appl. Mater. Interfaces.

[36] Lin W-H, Chiu Y-H, Shao P-W, Hsu Y-J (2016). Metal-particle-decorated ZnO nanocrystals: Photocatalysis and charge dynamics. ACS Appl. Mater. Interfaces.

[37] Atchudan R (2018). Highly fluorescent nitrogen-doped carbon dots derived from *Phyllanthus acidus* utilized as a fluorescent probe for label-free selective detection of Fe^3+^ ions, live cell imaging and fluorescent ink. Biosens. Bioelectron..

[38] Atchudan R (2018). Concurrent synthesis of nitrogen-doped carbon dots for cell imaging and ZnO@nitrogen-doped carbon sheets for photocatalytic degradation of methylene blue. J. Photochem. Photobiol. A.

[39] Kumar S, Sharma V, Bhattacharyya K, Krishnan V (2016). Synergetic effect of MoS_2_–RGO doping to enhance the photocatalytic performance of ZnO nanoparticles. New J. Chem..

[40] Wang S (2011). Temperature programmed precise control over the sizes of carbon nanospheres based on benzoxazine chemistry. J. Am. Chem. Soc..

[41] Zanni Elena, Chandraiahgari Chandrakanth, De Bellis Giovanni, Montereali Maria, Armiento Giovanna, Ballirano Paolo, Polimeni Antonella, Sarto Maria, Uccelletti Daniela (2016). Zinc Oxide Nanorods-Decorated Graphene Nanoplatelets: A Promising Antimicrobial Agent against the Cariogenic Bacterium Streptococcus mutans. Nanomaterials.

[42] Youssefa Z (2018). Dye-sensitized nanoparticles for heterogeneous photocatalysis: Cases studies with TiO_2_, ZnO, fullerene and graphene for water purification. Dyes Pigments..

[43] Sandoval A, Hernandez-Ventura C, Klimova TE (2017). Titanate nanotubes for removal of methylene blue dye by combined adsorption and photocatalysis. Fuel.

[44] Atchudan R (2018). One-pot dual product synthesis of hierarchical Co_3_O_4_@N-rGO for supercapacitors, N-GDs for label-free detection of metal ion and bio-imaging applications. Ceram. Int..

[45] Hong K (2014). Biomass derived hard carbon used as a high performance anode material for sodium ion batteries. J. Mater. Chem. A.

[46] Taha, K. K. *et al*. Green and sonogreen synthesis of zinc oxide nanoparticles for the photocatalytic degradation of methylene blue in water. *Nanotechnol. Environ. Eng*. **4** (2019).

[47] Hairom NHH, Mohammad AW, Ng LY, Kadhum AAH (2015). Utilization of self-synthesized ZnO nanoparticles in MPR for industrial dye wastewater treatment using NF and UF membrane. Desalin. Water Treat..

[48] Chen, X., Wu, Z., Liu, D. & Gao, Z. Preparation of ZnO photocatalyst for the efficient and rapid photocatalytic degradation of azo dyes. *Nanoscale Res. Lett*. **12** (2017). 10.1186/s11671-017-1904-4 PMC5319938 28235375

