# Supplementary material for: Fabrication of ZnO nanoparticles adorned nitrogen-doped carbon balls and their application in photodegradation of organic dyes
Source: Sci Rep. 2019 Dec 20;9:19509. doi: 10.1038/s41598-019-56109-3 (PMC6925138; doi:10.1038/s41598-019-56109-3)
Supplement: Supplementary file 1 — Supplementary Information [file 41598_2019_56109_MOESM1_ESM.doc]

**Fabrication of ZnO nanoparticles adorned nitrogen-doped carbon balls and their application in photodegradation of organic dyes**

Periyasamy Thirukumaran^a,1^, Raji Atchudan^a,1^, Asrafali Shakila Parveen^b,1^, Koteeswaran Kalaiarasan^c,1^, Yong Rok Lee^a^, Seong-Cheol Kim^a*^

*^a^School of Chemical Engineering, Yeungnam University, Gyeongsan 38541, Republic of Korea*

*^b^School of Material Science and Engineering, Myongji University, Yongin, Korea*

*^c^Center for Nanoscience and Technology, Anna University, Chennai, India*

^1^Authors contributed equally to this work

^*^Author for the Correspondence: E-mail: [sckim07@ynu.ac.kr](mailto:sckim07@ynu.ac.kr)

**Instrumentation methods**

The synthesized benzoxazine monomer and CBs were thoroughly characterized by various physicochemical techniques. Fourier transform infrared (FT-IR) spectra were obtained with a Perkin Elmer MB3000 FTIR spectrometer. The spectra were obtained at a resolution of 4cm^−1^ in the IR range of 400–4000cm^−1^. Samples were prepared by grinding with KBr and compressed to form discs. Nuclear magnetic resonance (NMR) spectrum was recorded by using an Agilent NMR, VNS600 at a proton frequency of 600MHz for 1H NMR. The solution was prepared by dissolving the sample in DMSO‑d6. X-ray powder diffraction (XRD) measurements were carried out using a PANalytical X’Pert3MRD diffractometer withmonochromatized Cu Kαradiation (λ= 1.54 Å) at 40 kV and 30 mAand were recorded in the range from 10 to 90° (2θ). The dry darkblackish-brown powder wasfilled in an XRD glass holder and was thenmeasured in reflection geometry. Raman spectrum was recorded on aThermo Scientific DXR SmartRaman spectrometer with the range from50 to 2500 cm−1. X-rayphotoelectron spectroscopy (XPS) spectra were achieved using a K-Alpha (Thermo Scientific). CasaXPS software was used for the decon-volution of the high-resolution XPS spectra. .Ultraviolet-visible (UV–vis) absorption spectra were recorded from 200to 800 nm using an OPTIZEN 3220UV spectrophotometer. Excitationand emissionfluorescence spectra were recorded using a Hitachi F-7000fluorescence spectrophotometer. The excitation wavelength wasvaried to determine the maximum emission intensity was achieved byvarying the excitation wavelength. The slit width wasfixed at 5 nm andthe scan speed was set to 240 nm/min.

**Photodegradation efficiency of bare ZnO nanoparticles**

**Figure S1** Degradation of MB dye in presence of bare ZnO nanoparticles under UV light (a) UV-Vis absorbance spectra at different time interval (min), (b) MB Degradation efficiency (%) and (c) Reaction kinetics
